# A step ahead: combining protein purification and correct folding selection

**DOI:** 10.1186/1475-2859-3-12

**Published:** 2004-10-07

**Authors:** Ario de Marco

**Affiliations:** 1 European Molecular Biology Laboratory, Meyerhofstr. 1, D-69117, Heidelberg, Germany

## Abstract

The success of recombinant protein expression seems unpredictable and even good yields of soluble proteins do not guarantee the correct folding. The search for soluble constructs can be performed by exploiting libraries and speeded up by automation, but these approaches are money and time consuming and the tags used for affinity purification can mask the real stability of the target proteins. The ideal purification protocol would include the structure quality control. A recent paper commented in this article describes a phage-display method to screen for antibodies that are able to re-fold after heat-denaturation and can be selectively affinity-purified only if monodispersed. It turned out that the proteins with high recovery performance after heat-shock were also suitable for efficient recombinant expression.

## Introduction

The possibility to produce recombinant proteins instead of recovering the native molecules offers the double advantage of higher yields and of a simplified purification protocol using affinity chromatography. At least half a dozen of the purification tags that have been proposed so far are routinely fused to the target proteins and used to perform affinity purification. *E. coli *is the most popular host for the expression of heterologous proteins but its simplified cell organization can be limiting for the expression of correctly folded recombinant proteins. No bioinformatic tools can predict if a construct will be expressed soluble in bacteria and, therefore, time-consuming cloning steps and expression optimization tests must be considered.

In most of the cases the affinity purification protocols are effective. However, the costs of the resins and proteases necessary to remove the tags can become a limiting factor. Furthermore, the different requirements for the chromatography steps and proteolysis make difficult to conceive automatic systems for obtaining purified homogeneous protein. As an alternative, we showed that the fusion of a target protein with a thermostable partner can be purified to homogeneity by heating [1]. Because the recovered target proteins resulted correctly folded only in some cases the method seems rather suitable for the preparation of antigens than for functionally active molecules.

We do not expect that heated proteins recover the native structural features but it is a common simplification to assume that a soluble protein is correctly folded and companies commercialize vectors with tags that "improve the solubility". Completely underestimated is the fact that a very soluble fusion partner can keep in solution unfolded target proteins. The work of the group around Travé showed the false results generated using fusion proteins and suggested a method for the evaluation of the aggregation state [2,3] and to consider the monodispersity as required parameter.

Therefore, methods would be envisaged that combine the purification to the selection for the correct folding. An important contribution in this direction is the recent paper published by Greg Winter's group [4].

## Discussion

The authors [4] describe a method for selecting antibody heavy chain variable regions resistant to the heat-induced aggregation. The antibody domains were displayed at the tip of filamentous bacteriophage and recovered by affinity binding to Protein A or to the specific antigen after the heating step. The purification was dependent on the correct folding of the antibodies since aggregates did not bind to the ligand. Once expressed in bacteria the selected antibodies showed a high yield and the property of reversible unfolding. In conclusion, the selection for the feature "re-folding from denatured/aggregation state" enabled the isolation of constructs adapted to recombinant expression. The results suggest that for the selected proteins the mechanisms leading to the re-folding into the native state are common to those that organize the folding of linear amino acid chains.

Functional genomic relies on the possibility of screening fast and efficiently large number of clones for their expression and correct folding. Several approaches have been suggested over the past years. An indirect method considers marker genes activated by misfolding [5] to discriminate aggregation-prone constructs. Otherwise, the solubility of reporter fusion partners has been considered [6,7]. Nevertheless, as well as for fusions with MBP or GST the correct folding of the fusion partner does not automatically mean that the target protein reached its native structure.

The elegance of the method described by Winter and co-workers relies on the use of a ligand that recognizes only the folded state of the protein to purify: the quality control is inclusive in the affinity purification. Furthermore, the phage-display format allows for the identification of the corresponding clone. As pointed out by the authors, such an approach is limited to those cases for which a conformation-dependent bait is available or, at least, a reliable method exists to discriminate between native and aggregated proteins. The logic of the experiment reminds me to the protocol used to select *in vivo*, directly and exclusively, for the class of conventional antibodies able to fold in the cytoplasm [8].

It is difficult to envisage a method applicable to all protein classes for selecting constructs that will express correctly folded proteins. Nevertheless, it is still possible to improve the yield of recombinant proteins that tend to aggregate. At least part of the unfolded proteins is not definitely trapped in aggregates. Re-solubilisation from bacterial inclusion bodies happens *in vivo *[9], the involvement of specific chaperones in the disaggregation process has been illustrated [10,11] and we used their co-expression to boost the bacteria re-folding machinery and increase the recombinant target protein yield [12]. Our unpublished data show that the chaperone-dependent solubilised protein is correctly folded, namely the recombinant chaperones are integrated into the *in vivo *protein quality control.

## Conclusions

The recombinant expression of proteins often induces the formation of soluble aggregates and several of such aggregates conserve sufficient features for being recovered by affinity chromatography. The simplification of having considered a purified soluble protein as a protein in native state has generated false results [2]. Therefore, methods that enable to selectively purify only correctly folded proteins [4] are welcome because couple purification and quality control. Unfortunately, their application is limited to few single protein classes for which a suitable binder exists. One important information of the Winter's group article is that the protein recovery after heat-shock, namely the possibility to re-fold correctly, correlates with correct folding in recombinant expression. Since the measurements of the aggregation index needs very small amounts of proteins [3] it would maybe worthy to screen using the aggregation index of heated domains to check if the Winter's group observation is a general rule and its application useful to select potentially soluble constructs in absence of specific binders.

Finally, the heat-selection can provide useful insights about the molecular features involved in the (re)-folding/disaggregation mechanisms.
